# Targeting the last polio sanctuaries with Directly Observed Oral Polio Vaccination (DOPV) in northern Nigeria, (2014–2016)

**DOI:** 10.1186/s12889-018-6182-2

**Published:** 2018-12-13

**Authors:** Charles Korir, Faisal Shuaib, Usman Adamu, Samuel Bawa, Audu Musa, Abba Bashir, Ayodeji Isiaka, Adamu Ningi, Charity Warigon, Banda Richard, Braka Fiona, Mkanda Pascal, Nkwogu Loveday, Sisay G. Tegegne, Mohammed Abdul-Aziz, Abdullahi Suleiman, Kabiru Mohammed, Melisa Corkum, Chima Onoka, Peter Nsubuga, Yared G. Yehualashet, Rui G. Vaz, Wondimagegnehu Alemu

**Affiliations:** 1 World Health Organization, Country Representative Office, Abuja, Nigeria; 2 National Primary Health Care Agency, Abuja, Nigeria; 3 World Health Organization, Regional Office for Africa, Brazzaville, Congo; 4 Global Public Health Solutions, Atlanta, GA USA; 5 UNICEF Country Office, Abuja, Nigeria

**Keywords:** Directly observed polio vaccination, Incentives, Supervision, And compliance

## Abstract

**Background:**

The declaration of poliomyelitis eradication as a programmatic emergency for global public health by the 65th World Health Assembly in 2012 necessitated innovations and strategies to achieve results. Review of the confirmed polio cases in 2013 showed that most of the cases were from non-compliant households, where parents connived with vaccinators to finger mark the children without actually vaccinating the children with oral polio vaccine or children were absent from home at the time of the visit of vaccinators.

**Methods:**

We used pre-post design to quantify the outcomes of directly observed vaccination in 90 local government areas from 12 northern Nigeria states at very high risk of polio transmission.

The strategy is an intervention, vaccinating children under the direct supervision of an independent supervisor to ensure compliance. Attractive incentives (pluses) were used to make parents willingly submit their children for vaccination or directly attract children to the vaccination teams or post as part of this strategy.

**Results:**

There was a steady increase in population immunity in all the 90 DOPV implementing LGAs since the introduction of DOPV in 2013. The number of states in which > 90% of children received > 4 OPV doses increased from 7 in 2013 to 11 states by July 2016.

Yobe state reported the highest proportional increase from 75 to 99% by July 2016 (22% increase), while Kano state reported 17% increase, from 82 to 99% by July 2016.

**Conclusion:**

Directly observed polio vaccination strategy improved uptake of polio vaccines and population immunity in high-risk areas for polio transmission.

## Background

The 65th World Health Assembly in 2012 declared poliomyelitis eradication as a programmatic emergency for global public health requiring the full implementation of current and new eradication strategies [1]. Nigeria responded by developing and implementing a national polio emergency plan to addresses the situation. Despite the significant improvement in supplemental immunization activities (SIAs) quality and remarkable reduction of 58% of polio cases in 2014 compared to 2013, the targets were not achieved due to various challenges responsible for periodic outbreaks within endemic countries and virus importations to non-endemic countries globally [2].

The Polio Eradication Initiative (PEI) faced numerous challenges in northern Nigeria; mainly noncompliance by parents and connivance of house to house vaccination team members with parents to fingermark the children without actual vaccination [3]. In-depth interview of caregivers during 60-day follow-up case investigations and supportive supervisory visit confirmed the assertion [4].

A review of the wild poliovirus (WPV) cases in 2014 showed that five out of the six cases were from noncompliant households. Furthermore, a review of independent monitoring data from previous rounds of SIAs showed that 70% of children continue to be missed due to child absent, and 12% due to non-compliance. The absent children were mainly in the streets, playground, schools, markets and social events such as naming ceremonies during the household visits by the teams [4].

The 28th expert review committee (ERC) on polio eradication and routine immunization in Nigeria recommended the scaling up of proven innovations in reaching the chronically missed children in the most high-risk areas [5].

Reports from previous SIAs have shown that transit vaccination teams can reach children from noncompliant families, those in the streets, markets, and social events with verifiable vaccinations outside, using attractive incentives (e.g. sweets, milk sachets, biscuits, soap). Outside vaccination ensures that children are physically observed receiving the vaccine, hence, the introduction of the directly observed polio vaccination (DOPV) on a large scale [6].

The DOPV processes facilitated the vaccination of children under the direct supervision of an independent supervisor to ensure compliance.

This study describes the rationale and processes used to implement directly observed polio vaccinations in Nigeria to improve uptake of polio vaccines in settlements with chronically missed children due to concealment of non-compliance by parents in connivance with fraudulent house to house vaccination team members.

## Methods

DOPV was conducted in 90 local government areas (LGAs) in 12 very high-risk states for polio in Nigeria, purposively selected based on the risk categorization by the National Emergency Operations Centre (EOC) and global goods classification [4]. In these LGAs, the wards (which is the next administrative level below the LGA) for implementation were identified based on the following criteria: densely populated settlements with reported high number of noncompliance, suspicious vaccination coverage, concealments of non-compliant cases, fraudulent house and finger- marking during the previous SIAs rounds. Also included are settlements with reported high numbers of pending unvaccinated children who were absent when the teams visited and many households with non-eligible children during previous vaccination team visits.

### Strategy

DOPV was implemented within the framework of the regular Immunization plus Days (IPDs). Exclusive outside vaccination was implemented in the first 2 days of the 6-day IPD exercise. The DOPV was conducted in streets, transit points, social and religious events. The exercise continued with the regular 4-day house to house vaccinations in conjunction with, transit and health camp teams, including revisits and resolving noncompliant cases. Mop-up vaccinations were conducted soon after the regular IPDs and all vaccination teams participated in reaching all pending households, working with traditional and religious leaders to resolve all pending non-compliance before the next round. All vaccinations during revisits and in non-compliance households were also done outside the household with direct observation by a supervisor.

### Community engagement

Members of the local government task force on immunization which comprise traditional leaders and other stakeholders were informed of the need to introduce verifiable vaccination under direct observation of supervisors. The introduction of the incentives to improve uptake of OPV vaccination, which included soap, milk sachets, sweet, noodle and sugar as the case may be was deliberated and agreed upon.

Furthermore, they accepted the introduction of these incentives and strategy in their communities and resolved to mobilize the communities to accept the incentives provided to children and caregivers during street vaccinations. The engagement of the traditional rulers raised community awareness and improved the credibility of polio vaccination processes amongst previously reluctant communities.

The pilot for this strategy was conducted during the August 2014 supplemental immunization plus days (SIPDs) in Ningi LGA of Bauchi state. This strategy was then scaled up to cover all the 11 high risk states following the recommendation of the 25th expert review committee meeting on Polio eradication and routine immunization [7].

### The DOPV teams

The DOPV team comprised one team supervisor (who was familiar with the daily route implementation plan from previous rounds of the SIAs), one OPV vaccinator, one community leader and one mobilizer with a megaphone, to further attract children in addition to the pluses.

In the security compromised areas of Borno State, the state EOC involved the community-based security vigilante, popularly known as Civilian Joint Task Force (CJTF) to provide security and crowd control to the vaccination teams.

### Scope of work

The DOPV teams developed their plans using the daily implementation plans and route maps used by the house-to-house vaccination teams. They also incorporated existing transit team’s micro-plans which focused on markets, motor parks, busy street junctions, border crossings, nomadic routes, water points, schools, churches and any other locations with high transit population. The DOPV team covered the catchment areas assigned to a house-to-house team in 2 days.

Each DOPV team was deployed to specific streets and transit points with the exact names of the sites of deployment during the 2-day outside vaccination. The teams did not enter any house during the 2 days of exclusive outside vaccinations but concentrated on attracting and mobilizing children and mothers with children < 2 years old for outside vaccination under observation of the independent supervisors.

### Supervision and monitoring

The role of the supervisor was to ensure vaccines are administered to children under observation to avoid malpractices such as finger marking without vaccination, inflating numbers of children vaccinated, and not adhering to the micro plans (i.e., not covering all streets and transit points). Each DOPV team was assigned a supervisor to observe activities and record same on a supervisory checklist. The team was also supervised at least twice per day by the ward focal person, field volunteers, and other senior supervisors using DOPV checklist and endorsing the team tally sheet (Table 1).

**Table 1:** Summary of Directly observed polio vaccination supervisory checklist, Jigawa and Zamfara states, September 2014 IPDs

S/No	Indicators	No. of DOPV teams	%
1	Team systematically moving and following the daily implementation plan	905	92.6
2	Local entertainment attracting people to the team	817	83.6
3	Community leader accompanying team	962	98.5
4	Team working in the assigned settlement according to plans	965	98.8
5	Adequate incentive (pluses) for the number of children seen	964	98.7
6	Children directly observed receiving OPV	964	98.7

### Pre-requisites for successful DOPV implementation

For a successful implementation, DOPV teams had to be provided with: i) adequate oral polio vaccines, ii) adequate attractive pluses, iii) good entertainment for children and very strong supervisory support.

## Results

**Fig. 1:**
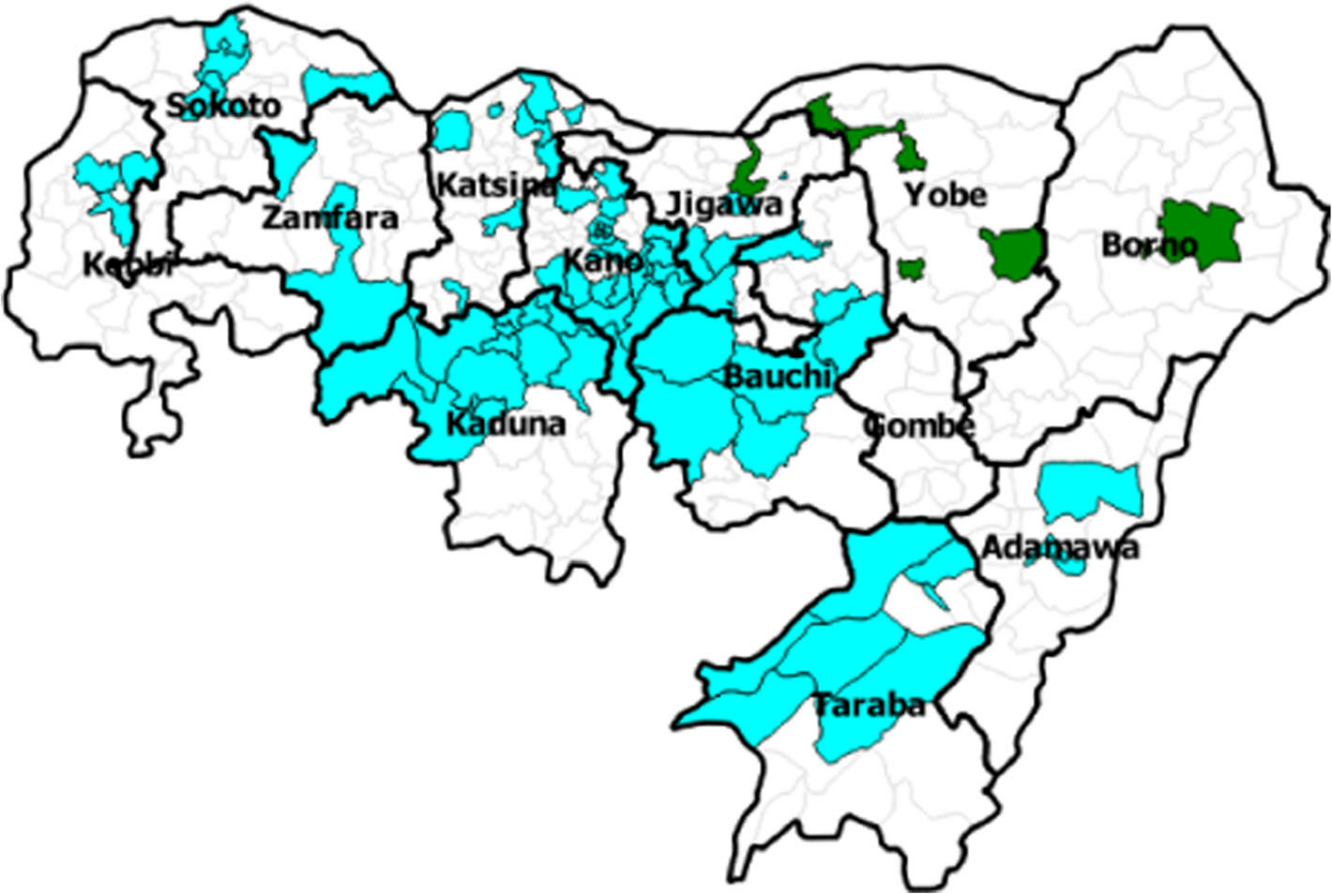
Geographical location of LGAs where DOPV was implemented – northern Nigeria, 2014–2016

Directly observed polio vaccination (DOPV) was conducted in 90 very high-risk LGAs of the 12 northern states (Adamawa, Bauchi, Borno, Jigawa, Kaduna, Kano, Katsina, Kebbi, Sokoto, Taraba, Yobe, and Zamfara) with very high risk for polio transmission. Figure 1 shows the geographic distribution of the LGAs where DOPV was implemented. Kano state had the highest number of LGAs (26), while Zamfara state had the lowest [2].

**Fig. 2:**
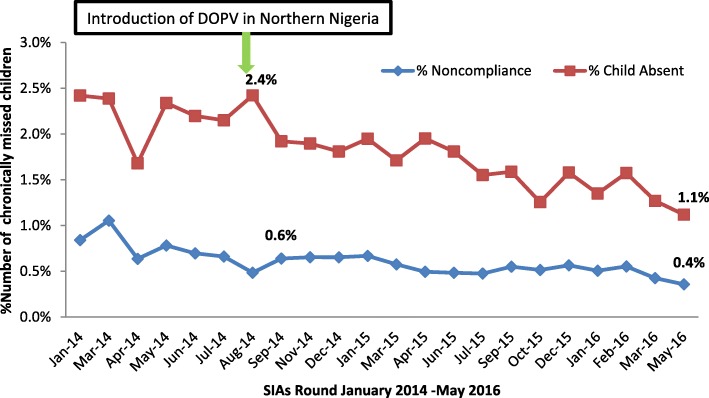
Trends of percentage missed children due to child absent and noncompliant households in the 90 DOPV LGAs in Northern Nigeria from January 2014 to May 2015

There was a decline in the percentage of missed children in the 90 LGAs from n 2014–16. Figure 2 shows that the missed children due to child absent declined from 2.4% in August 2014 to 1.1% in May 2016. Similarly, the missed children due to noncompliance declined from 0.6% in September 2014 to 0.4% in May 2016.

In Borno State, DOPV was used in the implementation of the outbreak response in four LGAs in June 2016. There was an increase in the absolute number of children vaccinated from 742,917 in May 2016 to 883,396 in July 2016. In July 2016, DOPV contributed 78.6% of the total number children vaccinated in all the four LGAs, with MMC reporting 85.5% of the children vaccinated through DOPV (Table 2).

**Table 2:** The impact of DOPV in reducing house to house team workload during IPDs, Limanti ward, MMC, Borno state, July 2016

Team Code	Total Households in Micro plan	No. of eligible children under five	No. of eligible children found already vaccinated during 2-day DOPV team	No. of eligible children vaccinated by house to house team	% Children found already vaccinated by DOPV team
239	120	111	92	19	82.9
240	147	139	120	19	86.3
241	121	141	123	18	87.2
242	124	218	199	19	91.3
243	117	137	119	18	86.9
244	110	194	175	19	90.2
245	132	241	204	37	84.6
246	125	201	183	18	91.0
247	131	141	122	19	86.5
248	130	254	235	19	92.5
249	132	230	212	18	92.2
Total	1389	2007	1784	223	88.9

The DOPV teams vaccinated 88.9% of the children in Limanti ward in 2 days, reducing the house-to-house vaccination team workload (Table 3). The proportion of the coverage by the DOPV team ranged from 82.9 to 92.5%.

**Table 3:** Trend of proportion of oral polio vaccine doses received by children with Non-Polio associated Acute Flaccid Paralysis (NPAFP) in Local Government Areas where DOPV was implemented, northern Nigeria, 2013–2016

State	LGAs	2013			2014			2015			2016		
		0 dose	1–3 doses	> 4 doses	0 dose	1–3 doses	> 4 doses	0 dose	1–3 doses	> 4 doses	0 dose	1–3 doses	> 4 doses
Yobe	4	4%	21%	75%	0%	9%	91%	0%	1%	99%	0%	3%	97%
Borno	4	7%	18%	75%	6%	19%	76%	2%	13%	85%	2%	12%	86%
Kano	26	2%	16%	82%	0%	4%	95%	0%	2%	98%	0%	1%	99%
Taraba	7	0%	12%	88%	0%	7%	93%	0%	8%	92%	0%	2%	99%
Adamawa	3	1%	11%	88%	1%	4%	95%	0%	4%	96%	0%	3%	97%
Sokoto	7	0%	9%	91%	0%	2%	98%	0%	1%	100%	0%	1%	100%
Bauchi	7	1%	9%	91%	0%	5%	94%	0%	5%	95%	0%	1%	99%
Zamfara	2	0%	9%	91%	0%	1%	99%	0%	0%	100%	0%	1%	99%
Kaduna	13	1%	9%	91%	0%	7%	93%	0%	4%	96%	0%	2%	98%
Jigawa	6	0%	5%	95%	0%	2%	98%	0%	2%	98%	0%	1%	99%
Katsina	8	2%	3%	95%	0%	0%	99%	0%	2%	98%	0%	1%	99%
Kebbi	3	0%	1%	99%	0%	1%	99%	0%	1%	100%	0%	0%	100%
Total	90	32 (1%)	252 (9%)	2588 (32%)	18 (1%)	133 (4%)	3281 (96%)	8 (0%)	140 (3%)	4902 (97%)	5 (0%)	69 (2%)	610 (93%)

**Fig. 3:**
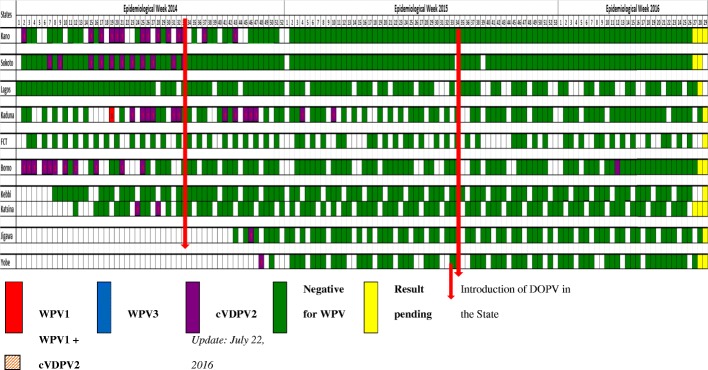
Trend of cVDPV from environmental surveillance: Weekly Polio statistics in Nigeria 2014–2016

There was a steady decline in the number cVDPV positive isolates from environmental surveillance. Figure 3 shows a decreased trend of cVDPVs from environmental sites in Kano and Sokoto after the introduction of DOPV in these states. The decline in Kaduna was slower, persisted for a short while before an eventual stop in epidemiologic week 10 of 2015. The weekly cVDPV isolation from environmental sites in Borno state stopped immediately after the introduction of DOPV in epidemiologic week 14.

Using the AFP surveillance performance as a proxy indicator for population immunity, the number of states with > 90% of non-polio AFP children receiving > 4 OPV doses increased steadily from 2013. This proportion increased from seven states (Bauchi, Kaduna, Sokoto, Zamfara, Jigawa, Katsina and Kebbi) to eleven by July 2016 (Table 4). Yobe state reported the highest proportional increase from 75% in 2013 to 99% by July 2016 (22% increase.) Over the same period, Kano state reported a 17% increase from 82% in 2013 to 99% in July 2016. In Borno State, despite the security challenges, the state reported an increase from 75% in 2013 to 86% by July 2016.

**Table 4:** Comparative cost per vaccinated child through DOPV and other interventions (house to house, Health camp, and transit vaccinations), Kaduna State, January 2016

Strategy	Total Children vaccinated (January 2015)	Cost of pluses (in Naira)	Cost of personnel	Cost of training	Cost of logistics	Other costs (Micro-planning, Social Mobilization, etc.)	Total Cost	Average cost per child in Naira	Average cost per child in USD^a^
DOPV	1,428,141	26,326,585	26,829,100	5,500,800	9,013,500	0	67,669,985	47.4	0.15
Other Strategies (House to House, Transit, Health camps)	2,293,756	0	65,968,900	2,677,050	43,391,950	61,786,892	173,824,792	75.8	0.24
Total	3,721,897	26,326,585	92,798,000	8,177,850	52,405,450	61,786,892	241,494,777	64.9	

^a^Official exchange rate (November 2016), 1USD = 315NGN

The cost per vaccinated child through DOPV was 0.15USD, compared with 0.24 USD for other interventions such as house to house and fixed post vaccinations. This cost was totaled for all the activities that were conducted for the various interventions (Table 5).

**Table 5:** Contribution of DOPV to the total number of children vaccinated <5Yrs in 4 LGAs, Borno State, from January to July 2016

LGA	January	February	May	June			July		
	No. of children Vaccinated	No. of children Vaccinated	No. of children Vaccinated	No. Vaccinated through DOPV	Total No. Vaccinated	% of Children Vaccinated through DOPV	No. Vaccinated through DOPV	Total No. Vaccinated	% .of children Vaccinated through DOPV
MMC	387,746	391,637	400,915	416,501	420,481	99.1	419,493	490,612	85.5
Jere	220,328	228,583	234,142	184,456	269,129	68.5	208,740	269,129	77.6
Mafa	32,409	33,265	34,555	26,584	36,332	73.2	31,433	40,656	77.3
Konduga^a^	39,846	41,575	73,305	0	75,531	0.0	35,788	82,999	43.1
Total	680,329	695,060	742,917	627,541	801,473	78.3	695,454	883,396	78.7

DOPV was implemented in 4 LGAs, June and July 2016 SIAs

^a^Note that only one ward out of 7 implemented DOPV in Konduga LGA in July 2016, due to insecurity

## Discussion

We found that the DOPV contributed to improved population immunity and interruption of poliovirus in the 90 high-risk LGAs with a record of immunity gaps in 12 northern states at risk of polio transmission in Nigeria. The strategy revealed that verifiable oral polio vaccinations given outside the household under the direct observation of an independent supervisor provided proof that OPV was administered to the recipient and there was compliance.

More than 20% of the eligible children in these very high-risk areas were given verifiable doses of the OPV. The study also revealed that the strategy has the potential of covering all the children in the catchment area if scaled up to the number of the days the regular SIAs are conducted with improved coverage and quality.

Our findings corroborate with studies where directly observed Ivermectin and Albendazole mass administration reported coverage rates between 75 and 85% multiple years of an administration with a compliance rate of about 88% [8]. The high-coverage in drug administration prevented and alleviated symptoms and morbidity on the one side, reduced transmission on the other, together with improving global health [9].

Implementation of directly observed oral polio vaccination required vigorous high-risk analysis of the area and adequate plans and engagement of community leaders and independent supervisors. The success was determined by the quality and drive of the supervisors coupled with ample supply of attractive child and adult pluses (incentives). Further, a daily implementation plan directing where and how the teams would move was essential, usually supported by the community leaders [10].

The impact of DOPV on SIAs quality is shown by the declining percentage of chronically missed children as reported by SIAs data. Lot Quality Assurance sampling (LQAs) surveys which are the gold standard in assessing IPDs quality in Nigeria also confirms the improvement in the number of LGAs accepted with coverage above 90% since the introduction of DOPV [11].

As acknowledged by the Independent Monitoring Board (IMB) report, Nigeria has achieved progress towards polio eradication, through a continual process of examining the problems and developing innovative solutions, among them; the directly observed polio vaccination (DOPV) [12],

In all the LGAs where DOPV has been consistently implemented since September 2014, we found that community leaders have reported an increase in acceptance to polio vaccinations as previously noncompliant parents now readily present their children for vaccinations owing to the attractive incentives given to eligible children and parents. With systematic continued engagement with key stakeholders, community leaders now give permission for vaccination of all the children found outside their homes even without their parents and caregivers.

While we have shown the role of DOPV in improving vaccinations coverage in northern Nigeria, the use of this innovation has limitations. First, although DOPV attracted very many children in settlements with noncompliance, some few chronic noncompliant households locked up their children preventing them from getting vaccinated outside. Secondly, the DOPV process is expensive considering the quantity of pluses required. However, detailed cost benefit analysis of using DOPV indicated that this strategy cost 0.15 USD per vaccinated child, compared with 0.24 USD for other interventions.

Despite these limitations, we observed that the use of DOPV coupled with other interventions rapidly reduced the number of missed children in the most high-risk LGAs leading to improvement in population immunity.

As polio eradication comes to a close, innovations such as DOPV that ensure quality and geographic reach should be used to target the last sanctuaries of active Polio transmission.

## Conclusions

Directly observed polio vaccination strategy improved uptake of polio vaccines resulting in increased population immunity in high-risk areas that were potential sanctuaries for polio transmission.

## Abbreviations

AFP: Acute Flaccid Paralysis
CJTF: Civilian Joint Task Force
cVDPV: Circulating Vaccine Derived Poliovirus
cVDPV2: Circulating Vaccine Derived Poliovirus2
DOPV: Directly observed polio vaccination
EOC: Emergency Operations Centre
EPI: Expanded Programme on Immunization
ERC: Expert Review Committee of Polio Eradication and Routine Immunization
IMB: Independent Monitoring Board
IPDs: Immunization Plus Days
LGA: Local Government Area
LQAS: Lot quality assurance sampling
MMC: Maiduguri Municipal Council
NPHCDA: National Primary Health Care Development Agency
OPV: Oral polio vaccine
PEI: Polio Eradication Initiative
SIAs: Supplemental Immunization Activities
SIPDs: Subnational Immunization Days
UNICEF: United Nations Children’s Fund
USD: United states Dollar
WHO: World Health Organization
WPV: Wild polio virus
